# Hematologic Abnormalities and Diseases Associated with Moderate-to-Marked Basophilia in a Large Cohort of Dogs

**DOI:** 10.3390/vetsci10120700

**Published:** 2023-12-12

**Authors:** Elizabeth Held, Hiroyuki Mochizuki

**Affiliations:** 1 Department of Public Health and Pathobiology, NC State College of Veterinary Medicine, Raleigh, NC 27607, USA; epheld@ncsu.edu; 2 Comparative Medicine Institute, North Carolina State University, Raleigh, NC 27606, USA

**Keywords:** basophilia, canine, eosinophilia, eosinophilic lung disease, heartworm, leukemia, myeloproliferative neoplasms

## Abstract

**Simple Summary:**

Our current understanding of the factors contributing to basophilia in dogs is limited primarily due to the absence of a large cohort study on this topic. To address this gap, our research aimed to retrospectively investigate hematologic abnormalities and specific conditions associated with moderate-to-marked basophilia in a large cohort of dogs. Our findings revealed that several common conditions were associated with moderate-to-marked basophilia in dogs, such as hypersensitivity disorders, parasitic infections, and neoplasia. Interestingly, dogs with marked basophilia were more likely to be diagnosed with neoplasia and had fewer inflammatory conditions than those with moderate basophilia. Additionally, when dogs with moderate-to-marked basophilia displayed concurrent eosinophilia, they were diagnosed more often with inflammatory conditions and less often with neoplasia. These results offer valuable insights for clinicians when they encounter dogs with significant basophilia. Depending on the extent of basophilia and the presence of concurrent eosinophilia, our findings can help clinicians make informed decisions regarding whether to prioritize investigating inflammatory disorders or neoplasia in these cases.

**Abstract:**

Basophilia is a rare hematologic finding in dogs. This research aimed to describe the hematologic and clinical characteristics of dogs with moderate-to-marked basophilia. CBC reports with blood smear examinations from dogs presented to the North Carolina State University Veterinary Teaching Hospital were retrospectively reviewed for basophilia (>193 cells/µL). We classified basophilia as moderate when counts were ≥500 cells/µL and marked when they reached ≥1000 cells/µL. We compared the hematologic and clinical profiles of dogs with moderate-to-marked basophilia (the basophilia group) to those without basophilia, serving as our control group. In addition, we investigated differences between dogs with marked basophilia versus those with moderate basophilia, as well as between dogs in the basophilia group with and without concurrent eosinophilia. Diseases associated with moderate-to-marked basophilia included eosinophilic lung disease (*p* < 0.0001), leukemia/myeloproliferative neoplasms (*p* = 0.004), parasitic infection (*p* = 0.004), mast cell tumor (*p* = 0.005), and inflammatory bowel disease (*p* = 0.02). Overall, dogs with marked basophilia had a lower frequency of inflammatory diseases (51% vs. 70%, *p* = 0.009) and a higher frequency of neoplastic diseases (48% vs. 26%, *p* = 0.003) compared to those with moderate basophilia. In the basophilia group, concurrent eosinophilia was only seen in 36% of dogs. Dogs with concurrent eosinophilia were more often diagnosed with inflammatory diseases (77% vs. 58%, *p* = 0.006), with fewer diagnoses of neoplasia (19% vs. 40%, *p* = 0.001), compared to dogs without concurrent eosinophilia. The findings of this study offer veterinary clinicians valuable guidance in determining diagnostic priorities for dogs with moderate-to-marked basophilia.

## 1. Introduction

Basophils are rare granulocytes that constitute less than 1% of circulating leukocytes in healthy mammals [1,2]. They originate from granulocyte-monocyte progenitors and mature into basophils primarily under the influence of interleukin 3 (IL-3) [3,4,5]. In the peripheral blood, basophils have a short half-life of approximately 6 h but can persist in tissues for up to 2 weeks when recruited in response to inflammatory mediators [4,5]. Basophils become activated through a wide range of molecules, such as immunoglobulins, various cytokines and chemokines, elements of the complement system, bacterial ligands, and proteases that interact with their surface receptors. Once activated, basophils release substances such as histamine, leukotriene C4, and a range of cytokines and chemokines that play a crucial role in both the early and late phases of the immune system response [6]. Basophils function as primary effector cells during parasitic infections and allergic inflammation and play a role in tumorigenesis and lipid metabolism [7,8].

In dogs, basophilia is a rare hematologic abnormality, often associated with IgE-mediated disorders and frequently occurring alongside eosinophilia [5]. In the veterinary literature, parasitism and allergic disease are reported to be the primary differentials for peripheral basophilia [5]. There are isolated reports of basophilia occurring in patients with eosinophilic airway disease [9] and with various neoplastic disorders such as basophilic leukemia [10], essential thrombocythemia [11], lymphomatoid granulomatosis [12], mast cell neoplasia [13], myelofibrosis [14], myeloid leukemias [5], and polycythemia vera [5].

Most of our current knowledge regarding basophils is derived from research in human medicine. Understanding basophil function and the factors contributing to basophilia in dogs remains limited, largely due to the scarcity of veterinary studies in this area. Thus, the objective of this study is to retrospectively outline the hematologic and clinical profiles of dogs with moderate-to-marked basophilia. We hypothesize that patients with moderate-to-marked basophilia will frequently exhibit concurrent eosinophilia. The prevailing co-occurring diseases are expected to align with those most commonly associated with eosinophilia, including mast cell tumors, parasitic infections, and hypersensitivity disorders.

## 2. Materials and Methods

### 2.1. Dogs with Moderate-to-Marked Basophilia (Basophilia Group)

We conducted a retrospective review of complete blood count (CBC) reports and blood smear examinations for all dogs presented to the North Carolina State University Veterinary Teaching Hospital during the period from 1 January 2010 to 31 December 2019, with a specific focus on identifying cases of basophilia (defined as having more than 193 cells/µL of basophils). Within this study, we categorized basophil counts ≥500 cells/µL and ≥1000 cells/µL as moderate and marked basophilia, respectively. Dogs falling into the moderate-to-marked basophilia categories were grouped together in what we referred to as the “basophilia group”.

### 2.2. Hospital-Based Control Dogs (Non-Basophilia Control Group)

Once the dogs in the basophilia group were identified, we established a hospital-based control group (referred to as the non-basophilia control group) by selecting one control dog for each dog in the basophilia group from the same time frame. The control dogs were selected based on the CBC identification number, which was given to each CBC analysis sequentially and in chronological order. For each dog in the basophilia group, we specifically chose a control dog with the immediately preceding CBC identification number and verified the absence of basophilia.

### 2.3. Hematologic Data

Automated CBC analyses were performed using the ADVIA 120 Hematology System (Siemens, Munich, Germany). For each automated CBC, a manual differential white blood cell (WBC) count was also performed. We retrieved specific CBC data, including spun packed cell volume (PCV), platelet count, WBC count, and counts of neutrophils, lymphocytes, monocytes, and eosinophils, from the medical records. When available, automated and/or manual reticulocyte count data were collected for anemic dogs. An absolute reticulocyte count >93,730/UL was defined as regenerative anemia. In addition, we investigated the possible connection between elevated blood cholesterol levels and basophilia in dogs, following anecdotal evidence suggesting a link between hyperlipidemia and basophilia [15]. Cholesterol values were retrieved from medical records if a concurrent chemistry panel had been performed within a few days of the CBC analysis. Cholesterol values were performed using the Cobas 6000 (Roche Diagnostics, Basel, Switzerland). To compare the basophilia group with the non-basophilia control group, we assessed factors such as cholesterol levels and hematologic abnormalities, including anemia, erythrocytosis, thrombocytosis, thrombocytopenia, leukocytosis, neutrophilia, neutropenia, eosinophilia, monocytosis, and lymphocytosis.

### 2.4. Clinical Data

Patient signalment information, including breed, age, sex, and neutering status, was gathered from electronic medical records. Clinical diagnoses of dogs in the basophilia and non-basophilia control groups were also obtained from electronic medical records. The diagnoses were determined by the attending clinicians in charge of the patient’s care. These clinical diagnoses were categorized into three disease categories, including inflammation, neoplasia, and other diseases.

### 2.5. Statistical Analysis

We compared categorical data, including the frequencies of hematologic abnormalities and disease prevalence between groups, using Fisher’s Exact Test. For numerical data with non-Gaussian distributions, we applied the Wilcoxon rank-sum test. To explore the correlation between basophil and eosinophil counts, we utilized the Pearson correlation coefficient. Statistical analyses were performed with JMP Pro software version 17. A *p*-value < 0.05 was considered significant.

### 2.6. Ethical Permit

No IACUC review was required, as the study reviewed existing CBC and blood smear data collected for diagnostic purposes from client-owned animals.

## 3. Results

### 3.1. Prevalence of Basophilia

Of a total of 64,156 canine CBCs that included blood smear evaluations, 1205 CBC reports (1.9%) revealed basophilia. Duplicate CBC reports were removed for individual dogs, for a total of 870 dogs with basophilia. Of these dogs, 646 (74.25%) showed mild basophilia, 159 (18.28%) displayed moderate basophilia with counts ranging from 500–999 cells/µL, and 65 (7.47%) exhibited marked basophilia with counts ≥1000 cells/µL. The basophilia group was therefore composed of 224 dogs with moderate-to-marked basophilia. Similarly, the non-basophilia control group also included 224 dogs, with one control dog matched for each patient in the basophilia group.

### 3.2. Patient Signalment

The two groups in this study—the basophilia group and the non-basophilia control group—were compared based on patient signalment. No statistically significant differences were observed with respect to age (*p* = 0.52). The median age of dogs in the basophilia and non-basophilia control groups was both 8 years (5 months to 18 years old and 9 months to 17 years old, respectively). The basophilia group consisted of 106 spayed females, 88 neutered males, 18 intact males, and 12 intact females, and the non-basophilia control group consisted of 106 spayed females, 104 neutered males, 11 intact males, and 3 intact females. There was no association between patient sex and group (*p* = 0.45), whereas intact animals were overrepresented in the basophilia group (*p* = 0.016). The basophilia group was composed of 10 mixed-breed dogs and 214 purebred dogs, consisting of 69 unique breeds. The most common purebred dog in the basophilia group was the Labrador Retriever (*n* = 24), followed by the German Shepherd (*n =* 14), Chihuahua (*n* = 13), Poodle (*n* = 7), American Staffordshire Terrier (*n* = 8), Boxer (*n* = 8), Golden Retriever (*n =* 7), Yorkshire Terrier (*n* = 6), Border Collie (*n* = 6), Beagle (*n* = 6), American Cocker Spaniel (*n* = 5), Siberian Husky (*n* = 5), Australian Shepherd (*n* = 5), and Jack Russell Terrier (*n* = 5). The remaining 95 purebred dogs consisted of 55 different breeds (each *n* < 5 and < 2% of the group). Similarly, the non-basophilia control group was composed of 13 mixed-breed dogs and 211 purebred dogs, consisting of 65 unique breeds. The most common breed in the non-basophilia control group was the Labrador Retriever (*n* = 20), followed by the Boxer (*n* = 13), Golden Retriever (*n* = 12), Maltese (*n* = 10), Poodle (*n* = 10), Dachshund (*n* = 9), Beagle (*n* = 8), German Shepherd (*n* = 7), American Cocker Spaniel (*n* = 6), Pug (*n* = 6), Jack Russell Terrier (*n* = 5), Australian Shepherd (*n* = 5), and Yorkshire terrier (*n* = 5). The remaining 95 purebred dogs consisted of 52 different breeds (each *n* < 5 and < 2% of the group). Among these purebred dogs, chihuahuas were overrepresented in the basophilia group (*n* = 13) compared to the non-basophilia control group (*n* = 3) (*p* = 0.02). Both groups had a similar number of Rottweilers (*n =* 4 in the basophilia group and *n* = 3 in the non-basophilia control group).

### 3.3. Hematologic Abnormalities

**Figure 1:**
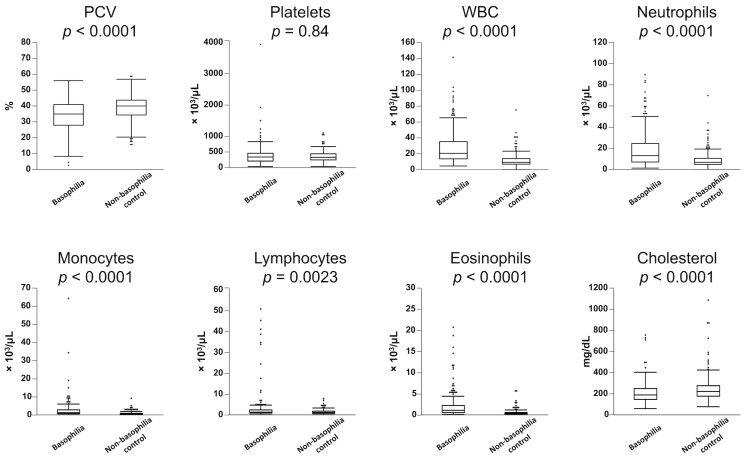
Comparison of hematological parameters between the 224 dogs with moderate-to-marked basophilia (basophilia group) and the 224 control dogs without basophilia (non-basophilia control group). Box plots indicate 25th to 75th percentiles, with whiskers indicating the minimum and maximum values within the 1.5 interquartile range and dots showing outliers.

Table 1 presents a statistical overview of the frequency of hematologic abnormalities in both the basophilia and non-basophilia control groups. Leukocytosis (83%), monocytosis (67%), neutrophilia (65%), anemia (54%), eosinophilia (36%), and lymphocytosis (12%) were more commonly observed in the basophilia group. Similarly, dogs in the basophilia group had significantly lower PCV and higher WBC counts, along with elevated counts of neutrophils, monocytes, lymphocytes, and eosinophils (Figure 1). There was a weak but significant correlation between basophil and eosinophil counts in the 224 dogs in the basophilia group (*r*^2^ = 0.29, *p* < 0.0001). In the basophilia group, 121 patients were anemic (64 non-regenerative, 34 regenerative). Reticulocyte counts were not performed in 23 of these patients. Anemia was further characterized as macrocytic hypochromic (*n* = 19), macrocytic normochromic (*n* = 3), normocytic normochromic (*n* = 54), normocytic hypochromic (*n* = 25), microcytic normochromic (*n* = 10), and microcytic hypochromic (*n* = 4). The remaining six patients exhibited an elevated MCHC, likely secondary to interfering substances (e.g., hemolysis, lipemia).

The frequency of hypercholesterolemia did not significantly differ between the basophilia group and non-basophilia control group (6% vs. 12%, respectively, *p* = 0.06); however, cholesterol concentrations were significantly lower in the basophilia group (Figure 1).

### 3.4. Specific Disease Associations

The common diseases observed in the basophilia and non-basophilia control groups are presented in Table 2. In the basophilia group, 145 dogs (65%) were diagnosed with inflammatory disease, 72 (32%) with neoplasia, and 7 (3%) with other disease processes. In the non-basophilia control group, 80 dogs (36%) were diagnosed with an inflammatory disease, 69 (31%) with neoplasia, and 75 (33%) with other disease processes. Inflammatory diseases occurred more frequently in the basophilia group (*p* < 0.0001), whereas both groups had a comparable number of patients diagnosed with neoplasia.

The common inflammatory conditions (*n* ≥ 5) observed in the basophilia group were eosinophilic lung disease, followed by parasitic infection, inflammatory bowel disease (IBD), immune-mediated hemolytic anemia (IMHA), bronchitis, gastrointestinal perforation/dehiscence, pancreatitis, and pemphigus foliaceus. Notably, only eosinophilic lung disease (*p* < 0.0001), parasitic infection (*p* = 0.004), and IBD (*p* = 0.02) were statistically more prevalent in the basophilia group compared to the non-basophilia control group. The parasites identified in the basophilia group were heartworm (*n* = 8), followed by hookworm (*n* = 4; one with coinfection with whipworm), caryospora (*n* = 1), and tapeworm (*n* = 1). Additionally, in the basophilia group, we observed four cases of non-bacterial infectious diseases, including aspergillosis (*n* = 2) and pythiosis (*n* = 2). In contrast, in the non-basophilia control group, the only parasitic infections identified were two cases of heartworm disease, and no other non-bacterial infections were observed.

The most common neoplastic conditions observed in the basophilia group were lymphoma, followed by mast cell tumors, and leukemia/other types of myeloproliferative neoplasms (MPNs). Only leukemia/MPNs and mast cell tumors were significantly overrepresented in the basophilia group. Types of leukemia/MPNs diagnosed included acute leukemias (*n* = 4; three unspecified and one of T-cell origin), T-cell chronic lymphocytic leukemia (*n* = 3), and essential thrombocythemia (*n* = 2). No cases of leukemias/MPNs were diagnosed in the non-basophilia control group. While the overall frequencies of lymphoma cases were comparable between the two groups, within the subset of immunophenotyped lymphomas, we observed a significant preponderance of T-cell lymphoma in the basophilia group (*p* = 0.03). T-cell lymphoma was more common in the basophilia group (13 out of 18 cases), with fewer B-cell lymphomas (5 out of 18). In contrast, B-cell lymphoma was more frequent (15 out of 24), with fewer T-cell lymphomas (9 out of 24) in the non-basophilia control group. Immunophenotyping was not performed in five and twelve lymphomas in the basophilia and non-basophilia control groups, respectively.

Chihuahuas were the only breed overrepresented in the basophilia group and were diagnosed with a variety of non-neoplastic diseases, including eosinophilic lung disease (*n* = 2), meningoencephalitis (*n* = 2), bronchitis (*n* = 2), cholangiohepatitis (*n* = 1), gastrointestinal (GI) perforation/dehiscence (*n* = 1), heartworm (1), immune-mediated thrombocytopenia (*n* = 1), IMHA (*n* = 1), GI ulcer (*n* = 1), and ocular trauma (*n* = 1).

### 3.5. Moderate versus Marked Basophilia

We compared patients with moderate basophilia and marked basophilia to determine if there were differences in hematologic abnormalities and disease associations based on the severity of basophilia. Thrombocytosis (30% vs. 15%, *p* = 0.01) and neutrophilia (75% vs. 61%, *p* = 0.045) were more commonly observed in dogs with marked basophilia compared to those with moderate basophilia. There was no statistical significance in the frequencies of other hematologic abnormalities (Table S1). Dogs with marked basophilia had higher WBC (*p* = 0.009) and eosinophil (*p* = 0.001) counts, whereas no statistical differences were observed in other hematologic parameters, including platelets (*p* = 0.17) and neutrophils (*p* = 0.16) (Figure S1).

Overall, dogs with marked basophilia had a lower frequency of inflammatory diseases (51% vs. 70%, *p* = 0.009) and a higher frequency of neoplastic diseases (48% vs. 26%, *p* = 0.003) compared to those with moderate basophilia (Table 3). Mesenchymal neoplasia was overrepresented in dogs with marked basophilia (*p* = 0.048). In addition, dogs with lymphoma tended to have marked basophilia (*p* = 0.051). In contrast, IMHA was observed only in dogs with moderate basophilia (*p* = 0.04).

### 3.6. Presence of Eosinophilia

In the basophilia group, we compared the frequencies of hematologic abnormalities and diseases between dogs with and without concurrent eosinophilia. Dogs with basophilia but without eosinophilia showed significantly increased incidences of anemia (63% vs. 38%, *p* = 0.0005) and thrombocytosis (23% vs. 12%, *p* = 0.049) and a decreased incidence of leukocytosis (79% vs. 91%, *p* = 0.02), compared to those with concurrent basophilia and eosinophilia. There was no statistical significance in the frequencies of other abnormalities between dogs with and without concurrent eosinophilia (Table S2). Similarly, dogs without concurrent eosinophilia in the basophilia group exhibited significantly lower PCV and higher counts of neutrophils, monocytes, and lymphocytes (Figure S2).

Table 4 presents a statistical overview of the frequencies of clinical diagnoses in the basophilia group, further stratified by the presence of concurrent eosinophilia. In the basophilia group, dogs with concurrent eosinophilia were more often diagnosed with inflammatory diseases (77% vs. 58%, *p* = 0.006) and fewer diagnoses of neoplasia (19% vs. 40%, *p* = 0.001) compared to dogs without concurrent eosinophilia. Dogs diagnosed with eosinophilic lung disease often had concurrent eosinophilia (*p* = 0.0007). Conversely, neoplasia was more often seen in dogs with basophilia and the absence of eosinophilia, particularly leukemia/MPNs (*p* = 0.03).

## 4. Discussion

Extensive research in human medicine has shed light on the critical roles of basophils in immunity, allergies, and cancer [8]. However, in dogs, little is known about the functions of basophils and the clinical significance of basophilia. This scarcity of information is, in part, due to the rarity of this hematologic abnormality in dogs. Complicating matters further, most automated hematological analyzers commonly used in veterinary practice cannot reliably detect basophils in dogs, necessitating the blood smear evaluation to evaluate basophilia [16]. In this study, we aimed to describe the hematologic and clinical characteristics of dogs with moderate-to-marked basophilia using a large dataset of canine CBC data with blood smear evaluation, enhancing our understanding of this rare yet significant hematologic abnormality in dogs. We also included hospital-based control dogs in our analysis to uncover overrepresented diseases associated with basophilia.

Our study identified an overrepresentation of Chihuahuas in the basophilia group. Although the specific cause for this observation remains uncertain, it is noteworthy that the Chihuahuas in this group were diagnosed with a range of inflammatory diseases, suggesting a predisposition in this breed towards inflammatory conditions. A previous study had suggested that Rottweilers tend to have higher circulating basophil counts [16]. However, in our investigation, we did not observe an increased risk of basophilia development among Rottweilers. This difference between our findings and the previous study could be attributed to various factors, including potential variations in the genetic makeup of Rottweilers in the United States compared to Sweden, as well as differing risks for developing basophilia in distinct geographic locations. These differences may arise from variations in the prevalence of pathogens (e.g., *D. repens*) and variances in preventive measures.

Our study revealed that basophilia is seldom an isolated condition and often co-occurs with other hematologic abnormalities. In particular, dogs in the basophilia group displayed a higher prevalence of monocytosis, neutrophilia, and eosinophilia compared to the non-basophilia control group. This association can be attributed to the increased frequency of inflammatory conditions diagnosed in the basophilia group. Additionally, we observed a greater incidence of anemia among dogs in the basophilia group. Many of these anemic cases were likely secondary to their underlying inflammatory conditions (i.e., anemia of inflammatory disease), particularly given that the degree of anemia was generally mild to moderate. As hyperlipidemia has been anecdotally linked to basophilia [15], our study examined the potential relationship between blood cholesterol concentration and basophilia in dogs. Surprisingly, we found the opposite result, with lower cholesterol concentrations in the basophilia group. Although the exact cause of this observation remains unclear, our data suggests that hypercholesterolemia is unlikely to be associated with basophilia in dogs. We could not explore the potential association between hypertriglyceridemia, another type of hyperlipidemia, and basophilia, as triglyceride measurements were not included in our chemistry panel and were unavailable for most dogs in both the basophilia group and non-basophilia control group.

In our study, eosinophilic lung disease was found to be the most frequent condition causing moderate-to-marked basophilia in dogs. Eosinophilic lung disease encompasses a group of disorders characterized by elevated eosinophil counts within the pulmonary airways and parenchyma [9,17]. Peripheral basophilia associated with eosinophilic lung disease in dogs has been previously reported, although the role of basophils in the development of this disease remains unclear [9,18]. In two studies, approximately 30% of dogs with eosinophilic lung disease presented with peripheral basophilia [9,18]. While the exact cause of eosinophilic lung disease remains unknown, it is believed to involve a significant Th2 immune response within the pulmonary airways and parenchyma [19]. Notably, basophils serve as one of the primary producers of IL-4, which is essential for the development of a Th2 response [20,21,22]. The presence of peripheral basophilia in dogs with eosinophilic lung disease further supports the involvement of a Th2 immune response in this condition. In addition to eosinophilic lung disease, another overrepresented inflammatory disease in the basophilia group was IBD. Given that basophils are associated with allergic inflammation, the presence of peripheral basophilia in these subsets of dogs with IBD may suggest an underlying allergic component contributing to IBD.

Our study confirmed that parasitic disease should be considered a differential diagnosis for dogs with moderate-to-marked basophilia, as commonly noted in the literature. Heartworm (*D. immitis*), hookworm (*Ancylostoma* sp.), and *Angiostrongylus* sp. infections have been documented in veterinary literature to cause peripheral basophilia [15,23,24]. In mice, basophils have a well-documented role in protection against parasites. Basophils can be stimulated through substances released from parasites or when their surface-bound IgE recognizes parasite-derived antigens [25]. Once basophils are activated, they release molecules such as histamine, proteases, and Th2-type cytokines that bolster the immune defense against parasites [25].

In humans, the most common neoplastic condition associated with basophilia is chronic myeloid leukemia (CML) [26]. However, in dogs, CML is uncommon, and when it does occur, basophilia is not consistently observed [27,28]. Basophilia has been reported in several dogs with leukemia/MPNs, including essential thrombocythemia [11], polycythemia vera [5], and basophilic leukemia [10]. In our study, we found that leukemia/MPNs were overrepresented in dogs with moderate-to-marked basophilia. Intriguingly, among the nine patients with leukemia/MPNs, four were further diagnosed with T-cell lymphoid leukemia. While there are no prior reports in the veterinary literature linking lymphoid leukemia to basophilia, there have been documented cases of T-cell lymphoma in cats resulting in marked paraneoplastic basophilia [29,30]. We also noted the preponderance of T-cell lymphoma in the basophilia group, suggesting a link between T-cell neoplasms and paraneoplastic basophilia. In cases of T-cell lymphoma, paraneoplastic eosinophilia has been more commonly observed than basophilia. This is thought to result from the overproduction of interleukin-5 (IL-5) by neoplastic Th2 lymphocytes [29]. In a similar vein, a comparable mechanism could be hypothesized in the context of basophilia, potentially involving IL-3, a critical growth factor for basophils. The validation of this hypothesis may be possible through the measurement of cytokines in dogs with lymphoid neoplasms and basophilia. We also confirmed the association between mast cell neoplasia and moderate-to-marked basophilia in our study, similar to the previous report [13].

Additionally, our study aimed to provide guidance on prioritizing specific diagnoses based on the extent of basophilia and the presence of concurrent eosinophilia. We discovered that patients with moderate basophilia were more frequently presented with inflammatory conditions, while those with marked basophilia were more commonly diagnosed with neoplasia. This pattern aligns with findings in human medicine, where patients with “reactive” or non-neoplastic basophilia typically do not exhibit basophil counts exceeding 1000 cells/µL [26].

Surprisingly, we found that only 36% of dogs in the basophilia group exhibited concurrent eosinophilia. This finding was unexpected, as it is commonly assumed that basophilia and eosinophilia typically coexist [5]. When concurrent eosinophilia was observed, inflammatory disorders were significantly more prevalent in the basophilia group. In contrast, when dogs presented with basophilia in the absence of eosinophilia, neoplasia was more frequently diagnosed.

This study comes with certain limitations that warrant consideration. First, our inclusion criteria focused on dogs with basophilia counts of ≥500 cells/µL in the basophilia group. This selection was made to concentrate on cases with more pronounced basophilia, aiming to identify associations with specific diseases. However, it is worth noting that additional statistically significant disease associations might have emerged if patients with milder basophilia were included. Second, as mentioned earlier, our study was unable to investigate any potential relationship between hypertriglyceridemia and basophilia. This was due to the absence of triglyceride measurements in our chemistry panel. A third limitation was that, due to the retrospective nature of the study, underlying parasitic infection could not be entirely ruled out in patients in the basophilia group diagnosed with other inflammatory and neoplastic conditions, as not every patient underwent extensive infectious disease testing. Future studies should address these limitations to further enhance our understanding of the underlying mechanisms responsible for basophilia in dogs.

## 5. Conclusions

Common conditions associated with basophilia in a large cohort of dogs were hypersensitivity disorders, parasitic infections, and neoplasia. Given our findings, it is important to rule out underlying neoplasia when a patient presents with marked basophilia as well as basophilia without accompanying eosinophilia. Conversely, inflammatory conditions may be prioritized when patients display moderate basophilia as well as basophilia with concurrent eosinophilia.

## Figures and Tables

**Table 1:** Frequencies of selected hematologic abnormalities and hypercholesterolemia in the basophilia group versus the non-basophilia control group.

Abnormalities	Basophilia Group (*n* = 224)		Non-Basophilia Control Group (*n* = 224)		
	Proportion	*n*	Proportion	*n*	*p* Value
Anemia	54%	121	31%	69	<0.0001
Erythrocytosis	0%	1	1%	3	0.62
Thrombocytosis *	22%	43	15%	31	0.12
Thrombocytopenia *	26%	52	19%	40	0.16
Leukocytosis	83%	187	34%	76	<0.0001
Neutrophilia	65%	146	35%	78	<0.0001
Neutropenia	2%	4	5%	11	0.11
Eosinophilia	36%	81	5%	12	<0.0001
Monocytosis	67%	149	30%	67	<0.0001
Lymphocytosis	12%	26	4%	9	0.004
Hypercholesterolemia **	6%	13	12%	24	0.06

* A platelet count was performed in 202 patients in the basophilia group and 206 in the non-basophilia control group. ** Cholesterol values were available in 201 patients in the basophilia group and 197 in the non-basophilia control group.

**Table 2:** Disease comparison in the basophilia group versus the non-basophilia control group.

Disease	Basophilia Group (*n* = 224)		Non-Basophilia Control Group (*n* = 224)		
	Proportion	*n*	Proportion	*n*	*p* Value
**Inflammation**	65%	145	36%	80	<0.0001
Eosinophilic lung disease	11%	25	0	0	<0.0001
Parasitic disease	6%	14	1%	2	0.004
IBD	6%	13	1%	3	0.02
IMHA	5%	11	3%	6	0.32
Bronchitis	3%	7	1%	2	0.18
GI perforation/dehiscence	3%	6	1%	2	0.29
Pancreatitis	2%	5	2%	4	1
Pemphigus foliaceus	2%	5	0%	0	0.06
Other inflammatory conditions	26%	59	27%	61	0.92
**Neoplasia**	32%	72	31%	69 *	0.84
Lymphoma	10%	23	16%	36	0.09
Mast cell tumor	9%	21	3%	6	0.005
Leukemia/MPNs	4%	9	0%	0	0.004
Mesenchymal neoplasia	4%	8	4%	9 *	1
Epithelial neoplasia	3%	7	7%	15 *	0.12
Other neoplasia	2%	4	2%	4	1
**Other**	3%	7	33%	75	<0.0001

* One dog was diagnosed with both biliary carcinoma and hemangiosarcoma and included in both mesenchymal and epithelial neoplasia.

**Table 3:** Disease comparison in dogs with marked versus moderate basophilia.

Disease	Marked Basophilia (*n* = 65)		Moderate Basophilia (*n* = 159)		
	Proportion	*n*	Proportion	*n*	*p* Value
**Inflammation**	51%	33	70%	112	0.009
Eosinophilic lung disease	15%	10	9%	15	0.24
Parasitic disease	3%	2	8%	12	0.36
IBD	5%	3	6%	10	0.76
IMHA	0%	0	7%	11	0.04
Bronchitis	2%	1	4%	6	0.68
GI perforation	3%	2	3%	4	1
Pancreatitis	2%	0	3%	5	0.33
Pemphigus foliaceus	3%	2	2%	3	0.63
Other inflammatory conditions	20%	13	29%	46	0.19
**Neoplasia**	48%	31	26%	41	0.003
Lymphoma	17%	11	8%	12	0.051
Mast cell tumor	9%	6	9%	15	1
Leukemia/MPNs	6%	4	3%	5	0.29
Mesenchymal neoplasia	8%	5	2%	3	0.048
Epithelial neoplasia	5%	3	3%	4	0.42
Other neoplasia	3%	2	3%	2	0.58
**Other**	2%	1	3%	6	0.68

**Table 4:** Disease comparison of dogs with concurrent eosinophilia in the basophilia group.

Disease	With Concurrent Eosinophilia (*n* = 81)		Without Concurrent Eosinophilia (*n* = 143)		
	Proportion	*n*	Proportion	*n*	*p* Value
**Inflammation**	77%	62	58%	83	0.006
Eosinophilic lung disease	21%	17	6%	8	0.0007
Parasitic disease	9%	7	5%	7	0.27
IBD	7%	6	5%	7	0.56
IMHA	1%	1	7%	10	0.06
Bronchitis	1%	1	4%	6	0.43
GI perforation	1%	1	3%	5	0.42
Pancreatitis	1%	1	3%	4	0.66
Pemphigus foliaceus	2%	2	2%	3	1
Other inflammatory conditions	32%	26	23%	33	0.16
**Neoplasia**	19%	15	40%	57	0.001
Lymphoma	6%	5	13%	18	0.17
Mast cell tumor	5%	4	12%	17	0.10
Leukemia/MPNs	0%	0	6%	9	0.03
Mesenchymal neoplasia	4%	3	3%	5	1
Epithelial neoplasia	4%	3	3%	4	0.71
Other neoplasia	0%	0	3%	4	0.30
**Other**	5%	4	2%	3	0.26

## Data Availability

The data presented in this study are contained within the article and in supplementary materials.

## Supplementary Materials

The following supporting information can be downloaded at: https://www.mdpi.com/article/10.3390/vetsci10120700/s1. Table S1: Frequencies of selected hematologic abnormalities and hypercholesterolemia in patients with marked versus moderate basophilia. Table S2: Frequencies of selected hematologic abnormalities and hypercholesterolemia in patients with and without concurrent eosinophilia. Figure S1: Comparison of hematological parameters between the dogs with moderate and marked basophilia. Figure S2: Comparison of hematological parameters between the dogs with and without concurrent eosinophilia in the basophilia group (dogs with moderate and marked basophilia).
