# Supplementary material for: Hematologic Abnormalities and Diseases Associated with Moderate-to-Marked Basophilia in a Large Cohort of Dogs
Source: Vet Sci. 2023 Dec 12;10(12):700. doi: 10.3390/vetsci10120700 (PMC10748300; doi:10.3390/vetsci10120700)
Supplement: Supplementary file 1 [file vetsci-10-00700-s001.zip › vetsci-2713951-supplementary.pdf]

Table S1. Frequencies of selected hematologic abnormalities and hypercholesterolemia in patients with marked versus moderate basophilia.

Abnormalities	Marked basophilia (*n* = 65)		Moderate basophilia (*n* = 159)		
	Proportion	*n*	Proportion	*n*	*p* value
Anemia	60%	39	52%	82	0.30
Erythrocytosis	2%	1	0%	0	0.29
Thrombocytosis	30%	19	15%	24	0.01
Thrombocytopenia	22%	14	24%	38	1
Leukocytosis	89%	58	81%	129	0.17
Neutrophilia	75%	49	61%	97	0.045
Neutropenia	3%	2	1%	2	0.58
Eosinophilia	46%	30	32%	51	0.07
Monocytosis	69%	45	65%	104	0.64
Lymphocytosis	9%	6	13%	20	0.65
Hypercholesterolemia	8%	5	5%	8	0.53

Table S2. Frequencies of selected hematologic abnormalities and hypercholesterolemia in the basophilia group with and without concurrent eosinophilia.

Abnormalities	With concurrent eosinophilia (*n* = 81)		Without concurrent eosinophilia (*n* = 143)		
	Proportion	*n*	Proportion	*n*	*p* value
Anemia	38%	31	63%	90	0.0005
Erythrocytosis	1%	1	0%	0	0.36
Thrombocytosis	12%	10	23%	33	0.049
Thrombocytopenia	22%	18	24%	34	0.87
Leukocytosis	91%	74	79%	113	0.02
Neutrophilia	57%	46	70%	100	0.06
Neutropenia	0%	0	3%	4	0.30
Monocytosis	58%	47	71%	102	0.06
Lymphocytosis	14%	11	10%	15	0.51
Hypercholesterolemia	4%	3	7%	10	0.38

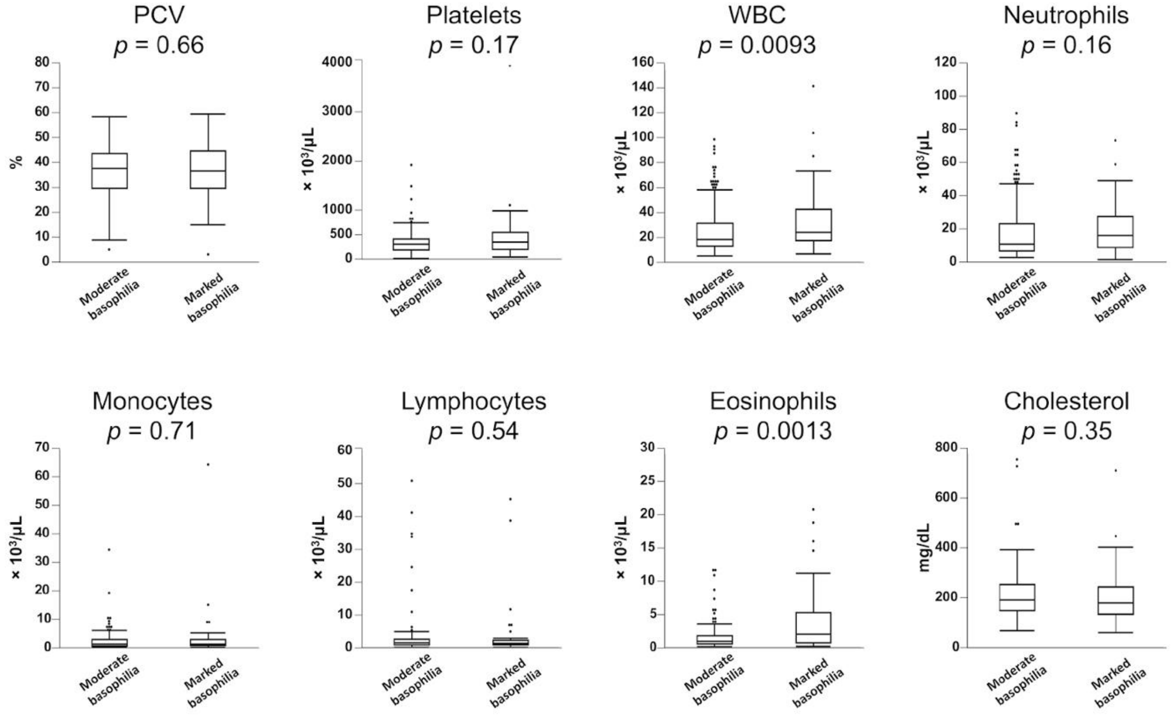

Figure S1. Comparison of hematological parameters between the dogs with moderate (n = 65) and marked (n = 159) basophilia. Box plots indicate 25th to 75th percentiles with whiskers indicating the minimum and maximum values within 1.5 interquartile range and with dots showing outliers.

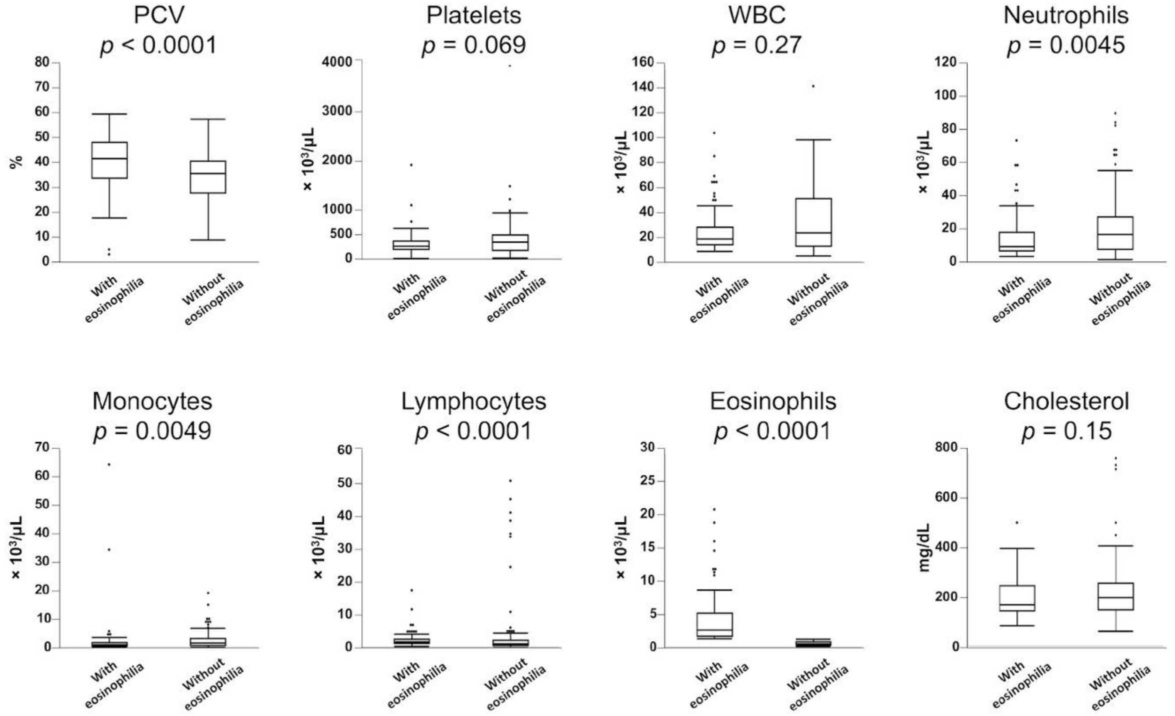

Figure S2. Comparison of hematological parameters between the dogs with (n = 81) and without concurrent eosinophilia (n = 143) in the basophilia group (dogs with moderate and marked basophilia). Box plots indicate 25th to 75th percentiles with whiskers indicating the minimum and maximum values within 1.5 interquartile range and with dots showing outliers.
